# Dynamic Parameter Calibration Framework for Opinion Dynamics Models

**DOI:** 10.3390/e24081112

**Published:** 2022-08-12

**Authors:** Jiefan Zhu, Yiping Yao, Wenjie Tang, Haoming Zhang

**Affiliations:** College of Systems Engineering, National University of Defense Technology, Changsha 410073, China

**Keywords:** opinion dynamics, data assimilation, public opinion, simulation calibration

## Abstract

In the past decade, various opinion dynamics models have been built to depict the evolutionary mechanism of opinions and use them to predict trends in public opinion. However, model-based predictions alone cannot eliminate the deviation caused by unforeseeable external factors, nor can they reduce the impact of the accumulated random error over time. To solve this problem, we propose a dynamic framework that combines a genetic algorithm and a particle filter algorithm to dynamically calibrate the parameters of the opinion dynamics model. First, we design a fitness function in accordance with public opinion and search for a set of model parameters that best match the initial observation. Second, with successive observations, we tracked the state of the opinion dynamic system by the average distribution of particles. We tested the framework by using several typical opinion dynamics models. The results demonstrate that the proposed method can dynamically calibrate the parameters of the opinion dynamics model to predict public opinion more accurately.

## 1. Introduction

Public opinion is the embodiment of the opinions, attitudes, and emotions expressed by the public, which affects multiple fields, such as human interactions, political orientation, financial policy, and even the military. Therefore, predicting the evolution trend of public opinion and providing valid explanations for its causes is a significant problem, both in theory and practice. Researchers have solved this problem by building opinion dynamics models. They simulate the interaction between individuals in society and changes in people’s opinions, which can effectively reveal the generation, diffusion, and aggregation of public opinion.

Faced with the problem of public opinion prediction and explanation, researchers have attempted to reveal and analyze the laws of the public opinion system by establishing opinion dynamics models. Earlier research in this field can be traced back to the French model proposed in 1956 and its subsequent French–Degroot model [1]. Since then, multiple opinion dynamics models have been proposed, which can be divided into two categories: discrete and continuous. Typical discrete opinion dynamics models include the Voter [2], the majority rule [3], and the Sznajd [4] models. The study of discrete opinion dynamics has become popular in recent years [5,6]. In these models, agent opinions have only two values: mostly “1” represents support and “−1” represents against. This makes them suitable for depicting either/or cases; for example, political election prediction [7,8]. However, they cannot distinguish between neutral and extreme opinions or situations that are more complicated. Continuous opinion dynamic models have solved this problem. The most famous among them are the Hegselmann–Krause (HK) model [9] and the Deffuant–Weisbuch (DW) model [10]. These two models also introduced an important concept of opinion dynamics, that is, the bounded confidence rule: individuals’ opinions are affected by others only when the difference between their own opinions and others’ opinions is less than a threshold [11]. Based on this research, researchers have proposed multiple extended models [12,13,14,15,16].

However, it is still difficult to predict public opinion using only dynamic opinion models. On the one hand, public opinion may be disturbed by unpredictable external factors, such as emergent public events or the speaking of Internet celebrities. On the other hand, because models are not the same as real systems, the result of model prediction will inevitably fail owing to the accumulation of random and systematic errors over time. Therefore, in addition to model construction, two questions need to be answered to use the opinion dynamics model to predict the evolution of public opinion.

**Problem 1** How to determine the initial values of the model parameters?

To solve this problem, the proposed framework determines the initial model parameters based on the genetic algorithm. We design a fitness function according to the characteristics of opinion dynamics and public opinion, which enables the framework to calibrate the parameters of the opinion dynamics models. Through selection, crossover, and mutation operations, we search for the parameters that best match the initial observation.

**Problem 2** How can parameters be adjusted dynamically when public opinion changes owing to external factors or errors? 

One feasible way to solve the above problem is to include empirical observation in opinion dynamics research to predict public opinion more accurately.

Machine learning is a popular data-driven method in multiple fields. Many studies have tried to predict the evolution of public opinion through machine learning methods [17,18,19,20,21,22,23]. However, compared with agent-based opinion dynamics models, machine learning cannot explain the inner mechanism of the system; it lacks interpretability and requires too much data to gain accuracy.

Traditional opinion dynamics models are mainly based on theoretical self-consistent and deductive analyses, which cannot be directly applied to predict public opinion. Recently, researchers have begun to import real social networks to test their models. Wang et al. [24] proposed an opinion dynamics analysis framework for a weighted directed complex network and imported a real email network to test their method. Zhu et al. [25] proposed an opinion dynamics model based on individuals’ attitude-hiding behaviors and simulated their model on real Epinions networks, which can well explain Duncan’s online social experiments [26]. However, these models are not yet associated with real observations; thus, they can only explain part of the public opinion phenomena and cannot calibrate the parameters of the models through simulation. 

Currently, some researchers attempt to introduce data obtained from real social networks to correlate theoretical models with empirical data. In most of their studies, a part of the data was selected as the training set to learn the parameters of the model, and the rest of the data were used as the test set to verify the prediction accuracy of the model. De et al. [27] used the spectral projected gradient method to maximize the likelihood function of the model parameters, thus realizing parameter calibration of their model. Xion [28,29] collected data from the product review websites Epinions and Ciao, randomly selected X% of the data as a training set to learn model parameters, obtain individual opinions, and model-related topic vectors. The remaining data were used as a test set to evaluate the accuracy of their model prediction. Moreover, Xiong gathered a large amount of data from Twitter and conducted sentiment analysis using part of the early data for parameter calibration and curve fitting; they then used the model to predict the remaining opinion dynamics and compared the results with the rest of the real data [30]. Johnson et al. [31,32] proposed an adapted genetic algorithm for modeling opinion diffusion and tested the method using the Degroot model with limited data. Kotisz [33] proposed a minimal opinion formation model that is flexible and can reproduce a wide variety of existing micro-influence assumptions and models. The model was calibrated using datasets gathered from real social networks [34,35]. Lu [36] collected large-scale data from Douban.com and expanded the Ising model to explore how individuals behave and the evolutionary mechanisms of their life cycles. The above studies introduced real data from social networks to fit their model parameters, but their calibration work was static; they did not calibrate their models with new observations. Monti et al. [37] proposed an inference mechanism that can fit the opinion dynamics model to the social trajectory in the real world, thereby increasing the ability to fit real data. However, in different social networks, user interaction behaviors and social trajectories may not be the same. Thus, it may not be suitable to apply the mechanism to other online social networks with different structures. In addition, compared to observing public opinion, collecting the social trajectories of users is more complicated. 

In summary, the field of opinion dynamics requires a dynamic calibration method that can be widely applied to all types of online social networks according to successive observations. 

Dynamic calibration is an important issue in the field of modeling and simulation. Recently, many researchers have explored dynamic calibration methods [38,39,40,41,42,43,44]. However, few have aimed at the field of opinion dynamics [45]. Thus, combining the advantages of the theoretical model and data-driven method to predict public opinion is a direction for future opinion dynamics research [46], and one way to achieve this is to introduce a data assimilation method. Common data assimilation algorithms include the Kalman filter, optimal interpolation, and the extended Kalman filter [47]. However, neither the Kalman filter nor the optimal interpolation method can deal with non-normally distributed and nonlinear systems. Furthermore, the extended Kalman filter requires that the probability distribution of the system be expressed by simple parameters, which is also not suitable for dealing with cases in opinion dynamics systems. Compared with other methods, a particle filter can be applied to nonlinear systems [48,49]. Moreover, the use of particle filters to realize data assimilation in discrete event simulation has also been proposed by researchers in recent years [50,51]. This indicates that a particle filter has the potential to be used in the prediction calibration of opinion dynamics.

Inspired by data assimilation methods, to predict the state of the changing opinion dynamic system, the framework uses a particle filter algorithm to calibrate the parameters of the models dynamically with successive observations. In the framework, we initialize the particles according to the results of the initial parameter calibration and determine the particle weights with successive observations. By continuously resampling the particles and updating the particle weights, the framework enables models to track the changing state of the opinion dynamic system by the average distribution of particles that hold the largest weights. 

We test the framework with four typical opinion dynamics models based on synthetic data, which simulate the changing public opinion systems. The results show that the framework can effectively determine the initial parameters of the models and dynamically calibrate the parameters of the opinion dynamics models over time, thus predicting public opinion more accurately. 

As illustrated above, in this study, the main contributions to the fields of opinion dynamics are as follows:

(1) We propose a framework that can dynamically calibrate the parameters of the opinion dynamics model to predict public opinion more accurately.
(2) We combine model prediction and empirical data into opinion dynamics research.
(3) We verified the effectiveness of the framework by simulation experiments.

The remainder of this paper is organized as follows. Section 2 provides the basis for our proposal. Section 3 describes the proposed framework in detail. Section 4 presents the simulation testing analysis. Section 5 provides a summary of this study.

## 2. Preliminary

To provide a basis for our proposal, we introduce some basic concepts of opinion dynamics and typical models, which will be used in the experiments in this study.

### 2.1. Brief Introduction of Opinion Dynamics

**Figure 1:**
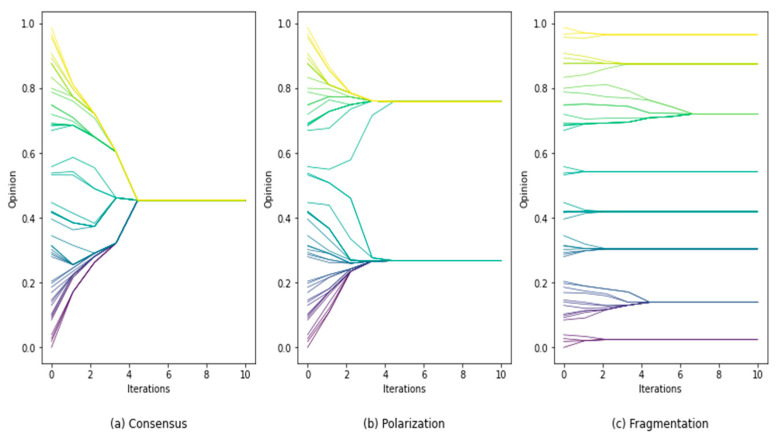
Evolution trends of opinion dynamics.

Opinion dynamics model the evolution process of agents’ opinions. Individuals in the system interact with others according to preset rules, and constantly update their opinions. As shown in Equation (1), let $x_{i}\left(t\right)$ be the opinion of individual i at time *t*. When an opinion dynamics system evolves to a steady state, it always shows an opinion distribution of consensus, polarization, or fragmentation, as shown in Figure 1 [52].

(1) $$x_{i}\left(t+1\right)=f\left(x_{1}\left(t\right),x_{2}\left(t\right)\ldots x_{j}\left(t\right)\right)$$

Generally, an agent’s opinion at the next moment is influenced by the opinions of other individuals. Individuals whose opinions influence each other can be regarded as having interactive relationships. The relationship between individual interactions can be depicted as a graph. Social networks can be regarded as a directed graph $G=\left(V,E\right)$, where V denotes the set of nodes (agents) in the network and E is the edge set formed by the connection of nodes, representing the interaction and influence relationship between individuals in the network.

### 2.2. Typical Opinion Dynamics Models

#### 2.2.1. Hegselmann–Krause Model 

The HK model [5] is one of the classic opinion dynamics models. The model is based on the bounded confidence rule; agents’ opinions are quantified as values ranging from 0 to 1, and $x_{i}\left(t\right)$ is the opinion of the *i*th agent at time *t*. Agent i only interacts with agent j whose opinion satisfies the condition $\left|x_{i}\left(t\right)-x\left(t\right)<\varepsilon \right|$. At each time step, individuals update their opinions as follows:

(2) $$x_{i}\left(t+1\right)=\frac{1}{N_{i}\left(t\right)}\underset{\left|x_{i}\left(t\right)-x_{j}\left(t\right)<\varepsilon \right|}{\sum }x_{i}\left(t\right)$$

#### 2.2.2. Expressed and Private Opinion Model 

The expressed and private opinion (EPO) model [7], proposed by Anderson, is an improvement of the classic FJ model [53,54]. In the simulation process, the model updates agents’ opinions using the following rules:

(3) $$x_{i}\left(t+1\right)=\lambda_{i}\left[a_{ii}x_{i}\left(t\right)+\overset{n}{\underset{j\neq i}{\sum }}a_{ij}{\hat{x}}_{j}\left(t\right)\right]+\left(1-\lambda_{i}\right)x_{i}\left(0\right)$$

(4) $${\hat{x}}_{i}\left(t\right)=\theta_{i}x_{i}\left(t\right)+\left(1-\theta_{i}\right){\hat{x}}_{avg}\left(t-1\right),$$

where $a_{ij}$ is the influence weight individual i has on individual j, $\lambda_{i},\theta_{i}$ denote agents’ stubbornness and adjustment coefficient, respectively. $x_{i}\left(t\right)$ is agent $A_{i}$’s private opinion at time *t* and ${\hat{x}}_{i}\left(k\right)$ is its expressed opinion at time *t*.

#### 2.2.3. Adapted Deffuant–Weisbuch Model

The adapted Deffuant–Weisbuch (ADW) model was proposed in 2022 [27] and is an adaptive model of the classic DW model. In the model, let $x_{i}\left(t\right)$ be individual i’s private opinion at time *t*, ${\hat{x}}_{i}\left(t\right)$ be its expressed opinion at time *t*, and $\mu_{i},p_{i}$ be related parameters for individuals. In the process of simulation, the model will randomly select two individuals in each iteration, if $|x_{i}\left(t\right)-{\hat{x}}_{j}\left(t\right)|<\epsilon$(ϵ is the bounded confidence threshold), individuals’ opinions will be updated by the following rules:

(5) $$x_{i}\left(t+1\right)=\left(1-\mu_{i}\right)x_{i}\left(t\right)+\mu_{i}{\hat{x}}_{j}\left(t\right)$$

(6) $${\hat{x}}_{i}\left(t+1\right)=\left(1-p_{i}\right)x_{i}\left(t+1\right)+p_{i}{\hat{x}}_{avg}\left(t\right)$$

#### 2.2.4. Attitude-Hiding Model

The attitude-hiding (AH) model was proposed in 2022 [10], which depicts online users’ behavior of hiding attitudes toward online social networks and can evolve multiple types of opinion dynamics.

In this model, agents estimate the popularity of their expressible opinions in their messages according to empirical knowledge. According to the outcome of their estimation, they decide whether to post messages as usual or to remain silent. Let$\;x_{i}\left(t\right)$ be agent $A_{i}$’s opinion at time *t* and $m_{i}\left(t\right)$ be the opinion $A_{i}$ wants to express at time *t*. For $A_{i}$’s fan $A_{j}$, the value of the feedback parameter$\;F_{j}=1$, 0 or −1, depending on whether $A_{j}$ likes, has no comment, or disagrees after $A_{j}$ receives $A_{i}$’s message with the opinion $m_{i}\left(t\right)$. Thus, the feedback $F_{j}$ is determined by

(7) $$F_{j}=\left\{\begin{array}{cc} 1, & \left|m_{i}\left(t\right)-x_{j}\left(t\right)\right|<\alpha \\ 0, & \alpha <\left|m_{i}\left(t\right)-x_{j}\left(t\right)\right|<\beta \;\\ -1, & \left|m_{i}\left(t\right)-x_{j}\left(t\right)\right|>\beta \end{array}\right.$$

where α is the boundary inside of which $A_{j}$ would give positive feedback ($F_{j}=1$), and β is the boundary outside of which $A_{j}$ would give negative feedback ($F_{j}=-1$).

Let $inN\left(i\right)$ be the set of $A_{i}$’s fans and let$\;\#inN\left(i\right)$ denote the number of$\;A_{i}$’s fans. $A_{i}$’s popularity of its messages to fans can be denoted by the feedback from its fans, which is

(8) $$R\left(m_{i}\left(t\right)\right)=\underset{j\in inN\left(i\right)}{\sum }F_{j}/\#inN\left(i\right)$$

If $\phi_{i}$ be a given threshold ($-1<\phi_{i}<1$), and if $R\left(m_{i}\left(t\right)\right)>\phi_{i}$, $A_{i}$ would believe that the potential gain from expressing opinions is large enough, it will express its opinion $m_{i}\left(t\right)$.

## 3. Dynamic Framework to Calibrate Opinion Dynamics Models

In this section, we introduce a detailed dynamic calibration framework for the prediction of opinion dynamics. Let the opinions of agents in the models be variables ranging from (0,1) and divide the opinion interval $\left[0,1\right]$ to K intervals uniformly. In this framework, we suppose that the observation of public opinion at time *t* is a vector with K dimensions $\vec{O_{t}}=\left\{o_{1},o_{2},\ldots ,o_{k}\right\}$, where $o_{i}$ is the distribution of all the opinions in the *i*th opinion interval. Let θ be the vector containing all parameters in opinion dynamics models and let the public opinion corresponding to the opinion dynamics system with θ at time *t* be a vector with K dimensions $\vec{M\theta_{t}}=\left\{m_{1},m_{2},\ldots ,m_{k}\right\}$, where $m_{i}$ is the distribution of all agents’ opinions in the *i*th opinion interval. 

Upon importing opinion dynamics models and initial observations into the framework, we first calibrate the model parameters based on the genetic algorithm, searching for a set of parameters with high adaptability to the initial data. Second, we conduct a particle filter based on the initial parameters obtained in the previous step and dynamically calibrate the model parameters with successive observations. The overall process of the framework is shown in Algorithm 1.
**Algorithm 1** Framework of calibrating the prediction for opinion dynamics models1:Initialize population; ⊳Population codes parameters of opinion dynamics models2:$\mathrm{Evaluate}\;\mathrm{population}\;\mathrm{by}\;\vec{O_{t_{0}}};$3:**while** the genetic algorithm method is searching **do**
4: Select population that best matches the observation;5: Crossover to generate new population, so as to search more efficiently;6: Mutate to realize local random search, and avoid unmature convergence;7: $\mathrm{Evaluate}\;\mathrm{Population}\;\mathrm{by}\;\vec{O_{t_{0}}}$;8:**end while**9:$\mathrm{Sample}\;\mathrm{particles}\;\mathrm{based}\;\mathrm{on}\;\vec{O_{t_{0}}}$; ⊳ Particles correspond to parameters of opinion dynamics models10:**If** obtain new observation **then** do11: Simulate opinion dynamics models according to the particles;12: Update particle weights;13: Estimate new system state through the average distribution of particles with the largest weights;14: Resample particles;15:**end if**

### 3.1. Initial Parameter Calibration Based on Genetic Algorithm

The genetic algorithm is a global search algorithm that is conducted through selection, crossover, and mutation operations to its generation during iterations, and evaluates its generation by calculating the fitness function. The proposed framework calibrates the initial parameters for the model based on the genetic algorithm and uses the results in the next step.

In this section, we introduce details of the method used in the framework. 

#### 3.1.1. Fitness Function

In this framework, individuals in the genetic algorithm correspond to a set of parameters of the opinion dynamics model. The smaller the deviation between the model output and initial data, the higher the fitness of the individual with the corresponding parameters. Let the fitness function *f* be the reciprocal of the total deviation between the model output and initial data as follows:

(9) $$f\left(\vec{M\theta_{t}},\vec{O_{t}}\right)=\frac{1}{\sum_{i=1}^{K}\left(\left|m_{i}-o_{i}\right|\right)}$$

#### 3.1.2. Selection

Individuals in the current population are selected with a certain probability for the next generation; this process is called selection. The probability of selection for the next generation depends on the fitness of the individual. The higher the fitness value, the higher the probability. In the proposed framework, we use one of the most popular selection methods, the roulette wheel selection method. In this method, the selection probability of each individual is proportional to its fitness value; the greater the fitness value, the greater the selection probability. Therefore, individuals with a large fitness value have a greater probability to be selected as a parent to generate a new individual. Let the probability of an individual $x_{i}$ be selected as $p\left(x_{i}\right)$, the fitness value of $\left(x_{i}\right)$ be $f\left(x_{i}\right)$, and the sum of all individuals’ fitness values be S; the roulette wheel selection method can be presented as follows:

(10) $$p\left(x_{i}\right)=\frac{f\left(x_{i}\right)}{S}.$$

#### 3.1.3. Crossover

The genetic algorithm maintains the diversity of the population through a crossover operator, which includes three steps. First, the number of individuals to be crossed is set according to the crossover probability, and then randomly select individuals to be exchanged. Second, the intersection position was randomly selected. Third, paired individuals exchange their corresponding attribute strings at the crossing position, thus forming new individuals. In this study, single-point crossing with a fixed crossover probability is used for the replication operation, which is performed by taking two chromosomes, dividing them at a randomly selected location, and swapping the right part, resulting in two different daughter chromosomes. For cases where selecting an intersection location has a certain meaning, single point crossover will cause less damage to the chromosomes.

#### 3.1.4. Mutation

In a genetic algorithm, the diversity of the population can be guaranteed by a mutation operation, which also prevents premature convergence. The mutation probability is small. In the mutation operation, first, the number of genes to be mutated is determined by the mutation probability, and then alleles to be reversed or replaced are randomly selected. 

We code the parameter values in binary form in the chromosomes, as shown below: 

Let the variable X to be optimized within the interval $\left[a,\;b\right]$, the number of genes be m, and the coding length be l, also the coding method is accurate to c decimal places, then:

(11) $$m=\log_{2}\left(b-a\right)+c\log_{2}10$$

(12) $$l=\left\{\begin{array}{cc} m & ,m\;is\;an\;integer\\ int\left(m\right)+1 & ,else \end{array}\right.$$

Therefore, the mutation is to select a certain location of the chromosome according to a fixed mutation probability and invert its value (0 becomes 1, 1 becomes 0), so as to enhance the random search ability of the genetic algorithm. Because the genetic algorithm can only search for one set of data and cannot retain historical information, the method introduced in Section 3.1 is a calibration method for determining initial parameters. We then combined more observations and used the data assimilation method to calibrate the models dynamically.

### 3.2. Dynamic Calibration Based on Particle Filters

In this step, we first express the probability distribution of the system state as a particle set. Particles are simulated to predict the prior distribution of the system state using the Monte Carlo method. We then merged real-time observations to calibrate the posterior distribution of the system state to ensure accurate analysis and prediction of the evolution of public opinion in social networks.

Let each particle have a set of parameters corresponding to the opinion dynamics model $\theta_{i},$ and let the state of the particle at time *t* be $\theta_{i}{}^{t}$. After parameter calibration, we obtained a set of optimal parameters for the initial observation, $\theta^{t_{0}}$. 

For a particle, let particle i’s initial parameter state be sampled from a Gaussian distribution, $\theta^{t_{0}}\sim N\left(\theta^{t_{0}},\sigma^{2}\right)$, where $\sigma^{2\;}$is the preset sampling deviation. Simultaneously, because of the nonlinear relationship between the opinion dynamics system and the output of the model with different parameters, we retain some of the particles whose initial state is sampled from a random distribution, $\theta^{t_{0}}\sim U\left(0,1\right)$. However, we assume that public opinion is in a steady state before being disturbed. Therefore, before obtaining the latest observation, the states of the particles remain the same, that is, $\theta_{i}{}^{t+1}=\theta_{i}{}^{t}$.

Let the state at time *t* be $S_{t}$, and the observation be $\vec{O_{t}}$. According to the Chapman–Kolmogorov equation, the prior probability density of $S_{t}$, $p(S_{t}|\vec{O_{1:t-1}})$ can be calculated as

(13) $$\left(S_{t}|\vec{O_{1:t-1}}\right)=\int p\left(S_{t}|S_{t-1}\right)p\left(S_{t-1}|\vec{O_{1:t-1}}\right)dS_{t-1},$$

where $p\left(S_{t}|S_{t-1}\right)$ represents the probability function of the state transition, which corresponds to the opinion dynamics model. Because we assume that the observed public opinion is in a steady state before $O_{t}$ is obtained, the models also output the results after they have evolved to a steady state. Therefore, $S_{t}=S_{t-1}$ before a new observation was obtained. The posterior probability density of $S_{t},\;p\left(S_{t}|\vec{O_{1:t}}\right)$ can be formulated as:

(14) $$p\left(S_{t}|\vec{O_{1:t}}\right)=\eta p\left(O_{t}|S_{t}\right),p\left(S_{t}|\vec{O_{1:t-1}}\right),$$

where η is the normalization coefficient, and the above equation represents the calibration process, which uses the likelihood $p\left(\vec{O_{t}}|S_{t}\right)$ between the state and observation to obtain the posterior distribution of states at the next moment $p\left(S_{t}|\vec{O_{1:t}}\right)$.

**Figure 2:**
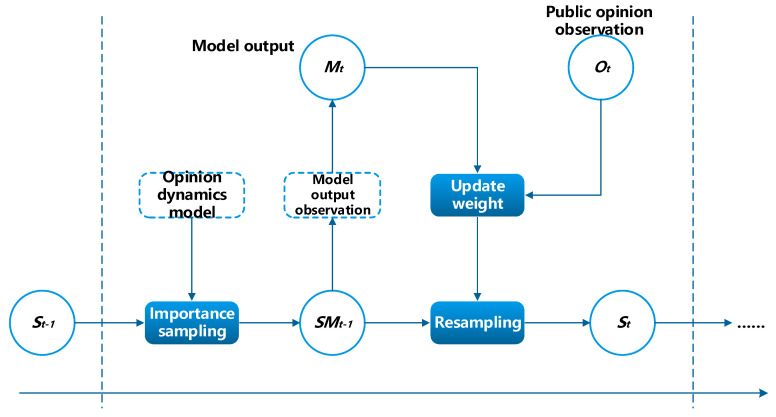
Data assimilation process for opinion dynamics based on particle filter.

Because it is difficult to obtain analytical solutions for nonlinear and non-Gaussian opinion dynamic systems, particle filters represent the posterior probability distribution of $S_{t}$ with a group of random samples (particles). When the number of particles is sufficiently large, the posterior probability of the system can be approximated sufficiently. The process of the proposed data assimilation method in the framework is illustrated in Figure 2.

In the particle filter, the posterior distribution is approximated by a set of particles $\left\{S_{t}^{k},w_{t}^{k}|k=1,\ldots ,N\right\}$. Here, $S_{t}^{i}$ represents the state of the ith particle and $SM_{t}$ is the model output when evolving to a steady state under the corresponding settings of each particle. $w_{t}^{i}$ is the weight of the ith particle. Each particle is a possible realization of a state; thus, the posterior distribution of the opinion dynamics system at time *t* can be represented as:

(15) $$P\left(S_{t}\right)\approx \overset{N}{\underset{k=1}{\sum }}w_{t}^{i}*\delta \left(S_{t}-S_{t}^{k}\right)$$

In the proposed framework, particles are a configuration set of the model, including the parameter setting and structure of the social interaction graph. Suppose $\left\{S_{t-1}^{k},w_{t-1}^{k}|k=1,\ldots ,N\right\}$ is known; then, the possible state at time *t* $SM_{t}^{k}$ can be inferred based on the opinion dynamics model; thus, newly generated sets of particles can be regarded as the particle representation of the prior distribution. Subsequently, each particle can calculate and update its weight according to the following observation:

(16) $${\hat{w}}_{t}^{k}=p\left(\vec{O_{t}}|S_{t}^{k}\right)*w_{t-1}^{k}$$

In detail, according to the calculation of the fitness function in Section 4.1, the closer the model output with parameters that correspond to a particle is to the observation, the greater the particle weight; thus, we have

(17) $$w_{i}{}^{t}\left(\vec{M\theta_{t}},\vec{O_{t}}\right)=\frac{1}{\sum_{i=1}^{K}\left(\left|m_{i}-o_{i}\right|\right)}$$

After several iterations, the estimated variance of the particle weight gradually increases, resulting in particle degradation; that is, the weight of most particles is too small for the particle set to effectively express the posterior probability distribution of the system state, and the simulation of these low-weight particles is not sufficiently meaningful. To this end, the method resamples the particles from the particle sets $\left\{SM_{t}^{k}|k=1,\ldots ,N\right\}$. Generally, the probability of selecting a particle is determined by its corresponding weight. Particles with high confidence are sampled more, which ensures that the particles converge to a state with high confidence and can obtain the posterior particle set $\left\{S_{t}^{k}|k=1,\ldots ,N\right\}$. After outputting the results, the framework continues to resample the particles. It retains the ν particles with the highest weights. Let the average state of those particles be $\theta^{t}{}_{avg}$, and the new states of other particles be sampled from a Gaussian distribution $N\left(\theta^{t}{}_{a\nu g},\sigma^{2}\right).$ Meanwhile, some particles are still selected, and their new states are sampled from the random distribution $U\left(0,1\right)$.

## 4. Simulation Tests and Analysis

In this section, we use the AH, ADW, EPO, and HK models to test the proposed dynamic calibration framework and solve the two problems. In Section 4.1, we calibrated the initial parameters of the four models based on synthetic datasets to verify the feasibility of the method proposed in Section 3.1. In Section 4.2, we solve the second problem, that is, assuming that the model is correct, how to calibrate the model as public opinion changes owing to external factors. Based on the results in Section 4.1, we initialized the particles and dynamically calibrated the four models using the particle filter method. The reason to use synthetic data is that the proposed framework is designed to solve the problem that, assume the opinion dynamics model is correct, how to dynamically calibrate the parameters with a new observation, so as to achieve more accurate model predictions. Whether the model can evolve specific real public opinion is not what this study considered. Compared with real public opinion data, synthetic data generated from opinion dynamics models fit better with corresponding models, which is easier to demonstrate whether the framework can dynamically calibrate model parameters to achieve more accurate predictions.

To quantitatively express the accuracy of the prediction and calibration, we propose the concept of deviation distance:

Let a node in the parameter space have an N-dimensional coordinate $\left(x_{1},x_{2},\ldots ,x_{n}\right)$, and the coordinates of the node that correspond to the actual parameters in the data are $\left({\hat{x}}_{1},{\hat{x}}_{2},\ldots ,{\hat{x}}_{n}\right)$. Then, the deviation distance was.

(18) $$Dev=\sqrt{\sum_{i=1}^{n}{\left(x_{i}-{\hat{x}}_{n}\right)}^{2}}$$

### 4.1. Calibration to Determine Initial Parameters

In this section, we search for the optimal initial parameters of the AH, ADW, EPO, and HK models using the parameter calibration method in the framework. For the initial observation, we set a combination of parameters for the four models and took the model output under these parameters as the synthetic initial observation.

For the AH model, we set ϱ=0.4, $\lambda_{i}=0.5$, $\phi_{i}=-0.5$, α=0.35, and β=0.4 to generate synthetic data. For the ADW model, we set $\mu_{i}=0.1$, $p_{i}=0.8$, and ϵ=0.3. For the EPO model, we set $\lambda_{i}=0.8$ and $\theta_{i}=0.3$. For the HK model, we set ε=0.1. The number of agents in the above models is 1000, and for those models that include a social network structure, we import a scale-free network with 1000 nodes. The initial opinions of the agents in the models were randomly sampled.

Calibrating the initial parameters for the four models, the results are shown in Table 1, Table 2, Table 3 and Table 4:

As shown in the tables, as the number of iterations increased, the deviation in the results for each model decreased. This indicates that the parameter calibration method in the proposed framework can infer the initial parameters of opinion dynamics models, thus answering Problem 1. The above results demonstrate the feasibility of the parameter calibration method and the reliability of the initial parameters for the models.

Note that the genetic algorithm may still obtain a local optimal solution instead of a global optimal solution. For example, in the results of the EPO model, the deviation with 100 iterations was greater than that with 50 iterations. A solution to this problem is to calibrate the model multiple times to avoid such situations.

Because prior knowledge cannot be fully used by the genetic algorithm to calibrate the model parameters with more observations, we then use the particle filter in the framework to dynamically calibrate the above models.

### 4.2. Dynamic Calibration with Successive Observation

First, we initialized the particles based on the results presented in Section 4.1. In the following experimental results, we present the particles with the largest weight in the parameter space.

In the experiments, synthetic data were generated after the models had evolved to a steady state under the preset parameters. The first group of data was the initial synthetic data used in Section 4.1. For the AH model, we changed ϱ, $\lambda_{i}$, and the remaining groups of data were generated with (ϱ,$\lambda_{i})=\left(0.8,0.2\right)$ and $\left(0.5,0.5\right)$. For the ADW model, we changed $\mu_{i}$, $p_{i}$, and the remaining data were generated with $(\mu_{i},p_{i})=\left(0.6,0.4\right)$ and $\left(0.3,0.5\right)$. For the EPO model, we changed $\mu_{i}$ and $p_{i}$, and the remaining data were generated with $\left(\mu_{i},p_{i}\right)=\left(0.5,0.5\right)$ and $\left(0.2,0.8\right)$. For the HK model, the data were generated with ε=0.2 and 0.05. The other parameters and configurations of the four models were the same as those in Section 4.1. Five hundred particles were used for the calibration of each model.

First, we present the results of the HK model. The results is shown in Table 5, Table 6 and Table 7. We selected 10 particles with the greatest weight as the results (the top 10 particle weights have been normalized), and it can be observed from the tables that, with the change in observed data, the parameters of the optimal particle can always be around the position corresponding to the real parameter.

However, note that the original HK model is mainly used to analyze the dynamic changes in opinion dynamics caused by different bounded confidence thresholds; the model is not built to fuse observations. Although the data assimilation algorithm proposed in this framework can be used to track and forecast self-generated synthetic data, the HK model may not be able to evolve some situations in public opinion due to the social network structure, other attributes of individuals, and even external influences.

**Figure 3:**
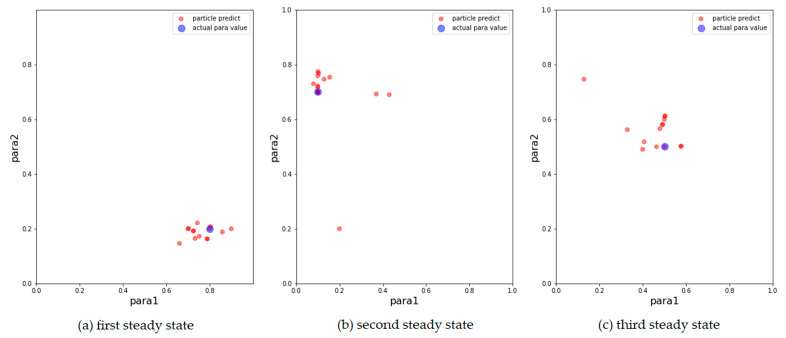
Results of AH model with 500 particles. The blue points are the parameters that generate synthetic data, the red points correspond to the 15 particles with the largest weight after filtering. (**a**) Results for the first observation. (**b**) Results for the second observation. (**c**) Results for the third observation.

**Figure 4:**
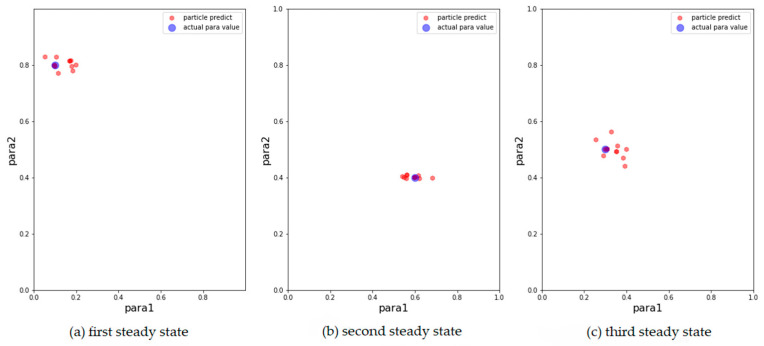
Results of ADW model with 500 particles. The blue points are the parameters that generate synthetic data, the red points correspond to the 15 particles with the largest weight after filtering. (**a**) Results for the first observation. (**b**) Results for the second observation. (**c**) Results for the third observation.

**Figure 5:**
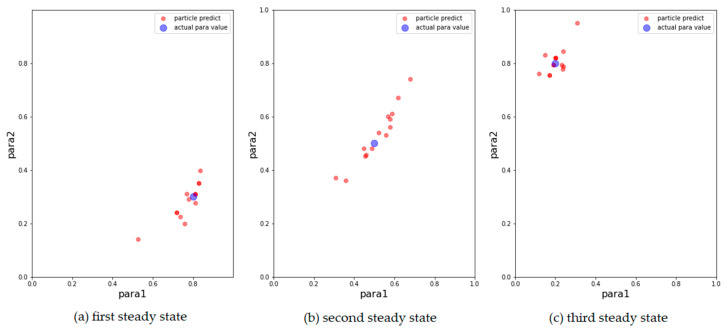
Results of EPO model with 500 particles. The blue points are the parameters that generate synthetic data, the red points correspond to the 15 particles with the largest weight after filtering. (**a**) Results for the first observation. (**b**) Results for the second observation. (**c**) Results for the third observation.

We then show the results of the AH, ADW, and EPO models in Figure 3, Figure 4 and Figure 5, respectively, on scatter plots in the parameter space. 

**Figure 6:**
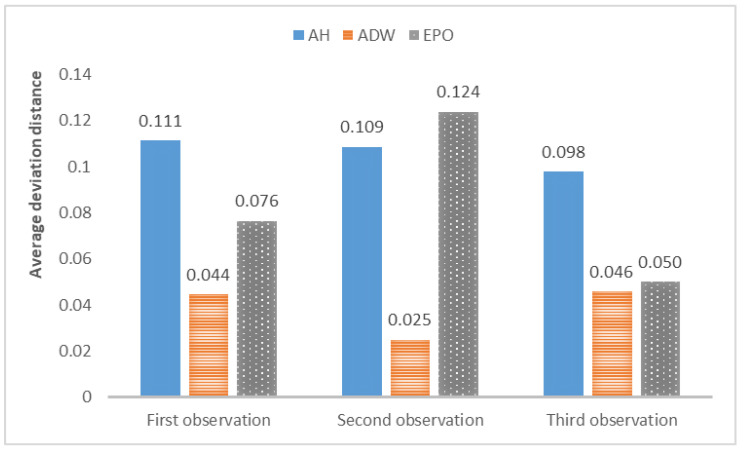
Deviation between the average position of the optimal particles after filtering and the position of the parameters for synthetic data.

The average deviation of the above results is shown in Figure 6.

From the above results, it can be concluded that the above models can track changes in the observations and dynamically calibrate the model prediction under the proposed framework. The positions of the calibrated particles in the parameter space can be approximately the positions of the parameters for the synthetic data. The results prove that the framework can dynamically calibrate the parameters of opinion dynamics models as public opinion changes and track the system through successive observations. Among the above models, the ADW model has the highest precision for calibration on synthetic datasets, whereas the distribution of particles of the EPO model for the second observation in Figure 5b is relatively dispersed. This may be because the ADW model is affected less by random factors than the EPO or the AH model. In addition, the results of the AH model are less accurate than the others, which may be due to its complexity. The AH model is built to predict real public opinion, and its mechanism is more complicated than that of the other models. Furthermore, a different set of parameters in the AH model may derive similar opinion dynamics, making particles that actually deviate from the real location significantly increase in weight, thus affecting the accuracy of the results. Nevertheless, opinion dynamics models with different parameters might show similar output results, leading to situations in which some particles in the above three experiments deviated more from the synthetic values. In future work, we will attempt to avoid this problem by improving the weight update rules.

## 5. Conclusions

In this study, a dynamic framework is proposed to calibrate the parameters of opinion dynamics models to predict public opinion more accurately. The framework does not require detailed observation from individuals’ social traces or activity records, but the overall public opinion, which is easier to obtain comparing with others’ work. The simulation results for the AH, ADW, EPO, and HK models show that the framework can be applied to multiple opinion dynamics models without changing the structure of the model, integrating model output and observation, and dynamically calibrating model parameters. However, due to time constraints, we did not test the framework with more particles on multi-dimension parameters for opinion dynamics models. In future work we will further improve the proposed framework by developing data crawling and sentiment analysis methods to make a prediction system. In addition, we will try to adjust the algorithm to achieve a parallel mechanism (both a genetic algorithm and a particle filter algorithm are suitable for a parallel algorithm) and use high-performance computing to set more particles and populations for simulation, so as to achieve more rapid and accurate prediction results with good interpretation. Lastly, we will attempt to obtain real public opinion data and improve the weight update rule and sampling strategy in the framework to support opinion dynamics models to predict real public opinion.

## Figures and Tables

**Table 1:** Results of parameter calibration for AH model.

	AH Model			
Number of iterations	50	100	1000	Actual value
para1	0.685	0.750	0.786	0.8
para2	0.787	0.197	0.204	0.2
Deviation	0.598	0.050	0.015	

**Table 2:** Results of parameter calibration for ADW model.

	ADW Model			
Number of iterations	50	100	1000	Actual value
para1	0.228	0.165	0.141	0.1
para2	0.826	0.795	0.796	0.8
Deviation	0.131	0.065	0.041	

**Table 3:** Results of parameter calibration for EPO model.

	EPO Model			
Number of iterations	50	100	1000	Actual value
para1	0.85	0.866	0.787	0.8
para2	0.354	0.574	0.330	0.3
Deviation	0.071	0.282	0.033	

**Table 4:** Results of parameter calibration for HK model.

	HK Model			
Number of iterations	50	100	1000	Actual value
para1	0.112	0.100	0.099	0.1
Deviation	0.012	0	0.001	

**Table 5:** Results when the public opinion system reaches a steady state for the first time (parameter value for the synthetic data is 0.1).

Actual Value	Particle Value	Particle Weight	Deviation
0.1	0.100	0.175	0.001
	0.100	0.175	0.001
	0.097	0.113	0.027
	0.102	0.104	0.019
	0.097	0.098	0.029
	0.104	0.073	0.038
	0.104	0.073	0.038
	0.100	0.064	0.003
	0.095	0.063	0.046
	0.094	0.062	0.057

**Table 6:** Results when the public opinion system reaches a steady state for the second time (parameter value for the synthetic data is 0.2).

Actual Value	Particle Value	Particle Weight	Deviation
0.2	0.180	0.148	0.097
	0.180	0.148	0.099
	0.180	0.114	0.098
	0.181	0.113	0.097
	0.222	0.110	0.112
	0.255	0.107	0.275
	0.185	0.078	0.074
	0.158	0.061	0.208
	0.162	0.061	0.190
	0.192	0.059	0.040

**Table 7:** Results when the public opinion system reaches a steady state for the third time (parameter value for the synthetic data is 0.05).

Actual Value	Particle Value	Particle Weight	Deviation
0.05	0.050	0.195	0.003
	0.049	0.110	0.010
	0.049	0.110	0.010
	0.049	0.110	0.010
	0.049	0.110	0.010
	0.049	0.110	0.010
	0.052	0.066	0.018
	0.048	0.066	0.018
	0.048	0.066	0.018
	0.053	0.059	0.032
